# Exerkines in diabetic retinopathy: from mechanisms to therapeutic prospects

**DOI:** 10.3389/fendo.2026.1762255

**Published:** 2026-03-10

**Authors:** Juming Zhu

**Affiliations:** Department of Ophthalmology, The First People’s Hospital of Yancheng, The Yancheng Clinical College of Xuzhou Medical University, Yancheng, Jiangsu, China

**Keywords:** exerkines, diabetic retinopathy, anti-inflammatory effect, antioxidant, neuroprotection, therapeutic strategies

## Abstract

Diabetic retinopathy (DR) is a leading cause of vision loss worldwide, driven by chronic metabolic dysregulation that promotes inflammation, oxidative stress, and progressive neurovascular unit dysfunction in the retina. While regular exercise is an effective non-pharmacological strategy to reduce diabetes-related complications, accumulating evidence suggests that its retinal benefits extend beyond systemic metabolic control and are mediated in part by exercise-induced bioactive factors known as exerkines. Secreted from skeletal muscle, adipose tissue, liver, and other organs, exerkines act as endocrine signals linking physical activity to tissue-specific adaptations. This review provides a retina-focused, cell-type-oriented synthesis of current evidence implicating key exercise-responsive exerkines, including irisin, adiponectin, brain-derived neurotrophic factor, fibroblast growth factor-21, apelin, and clusterin, in pathways relevant to DR pathogenesis. We systematically map reported exerkine actions to retinal endothelial cells, pericytes, Müller glia, microglia, neurons, and the retinal pigment epithelium, while explicitly distinguishing findings from retinal or DR-specific models from those extrapolated from extra-ocular systems. We further integrate emerging data on exercise modality-specific exerkine signatures and discuss their translational relevance, limitations, and safety considerations across different stages of DR. In total, this review highlights exerkines as candidate mediators and biomarkers of exercise-retina crosstalk and outlines priorities for mechanistic validation and clinical translation alongside established therapies such as anti-VEGF treatment.

## Introduction

Diabetic retinopathy (DR) remains a leading cause of vision loss worldwide. Among the estimated 463 million adults living with diabetes, approximately 170 million exhibit some degree of DR, reflecting both the rising global burden of diabetes and persistent limitations in disease prevention and modification (1–4). Despite advances in screening and treatment, substantial regional and demographic disparities remain. While systematic screening in high-income regions has stabilized prevalence, DR continues to account for significant visual impairment (2, 5). In low- and middle-income countries, limited healthcare resources and inadequate screening contribute to disproportionately higher disease burdens (6–8). Over a lifetime, DR develops in 50–60% of individuals with type 2 diabetes and up to 90% of those with type 1 diabetes, with older age and male sex conferring additional risk (2, 9, 10).

Clinically, early non-proliferative DR (NPDR) is characterized by microaneurysms, hemorrhages, and exudates, progressing toward widespread vascular dysfunction and ischemia (11). Advanced proliferative DR (PDR) involves pathological neovascularization, retinal pigment epithelium (RPE) migration, and fibrovascular membrane formation, often resulting in vitreous hemorrhage and tractional retinal detachment with a high risk of irreversible vision loss (12–15). Current management strategies, including intravitreal anti-VEGF (vascular endothelial growth factor) therapy, corticosteroid implants, laser photocoagulation, and pars plana vitrectomy, have improved short- to mid-term visual outcomes and reduced complications in many patients (16–19). However, these approaches often require repeated invasive procedures and substantial resources, and they largely target late-stage vascular manifestations rather than fully restoring long-term retinal vascular integrity, neurovascular coupling, and neuronal function; incomplete response/nonresponse and persistent neurovascular unit dysfunction remain important limitations (12, 13, 20).

Reviews focused on physical activity and DR predominantly emphasize epidemiological associations between exercise levels and DR prevalence or severity, with limited mechanistic resolution linking systemic exercise adaptations to specific retinal cell types or signaling pathways (21, 22). Exerkine-centered reviews comprehensively catalog exercise-responsive cytokines, myokines, hepatokines, adipokines, metabolites, and extracellular vesicles (EVs), highlighting their cardiometabolic benefits in diabetes, yet rarely interrogate their direct actions on the retinal neurovascular unit or their relevance to microvascular complications (23–25). Conversely, mechanistic DR reviews primarily focus on locally acting growth factors and inflammatory mediators, such as VEGF, Tumor Necrosis Factor-α (TNF-α), Interleukin-1 beta (IL-1β), Interleukin-6 (IL-6), and adhesion molecules, that govern vascular permeability, leukostasis, and pathological neovascularization, with comparatively little consideration of exercise-responsive endocrine or EV-mediated signaling as upstream, modifiable regulators (26, 27). Although emerging work acknowledges exercise-driven inter-organ crosstalk and EV-mediated communication, a unified framework integrating exercise, circulating exerkines/EV cargos, retinal cell-specific targets, and DR phenotypes is still lacking (28–31). Addressing this gap may provide a mechanistic basis linking lifestyle interventions to molecular pathways governing retinal inflammation, angiogenesis, and neurovascular dysfunction in diabetes.

In this review, we integrate these perspectives by synthesizing current evidence on exercise-responsive exerkines within the established cellular framework of DR. We emphasize cell-type-aware mechanisms across key retinal compartments, consider exercise modality-dependent exerkine signatures, and outline translational pathways linking exercise adaptation to biomarkers and exerkine-based augmentation strategies. Exerkines such as irisin, adiponectin, fibroblast growth factor-21 (FGF21), apelin, and lactate regulate angiogenesis, endothelial permeability, inflammation, and neuronal survival, processes central to DR progression (24, 32, 33). By integrating mechanistic and translational insights, this work aims to clarify how exercise-informed strategies may complement existing therapies and improve long-term outcomes in diabetic retinopathy.

## Pathogenic mechanisms of DR

**Figure 1:**
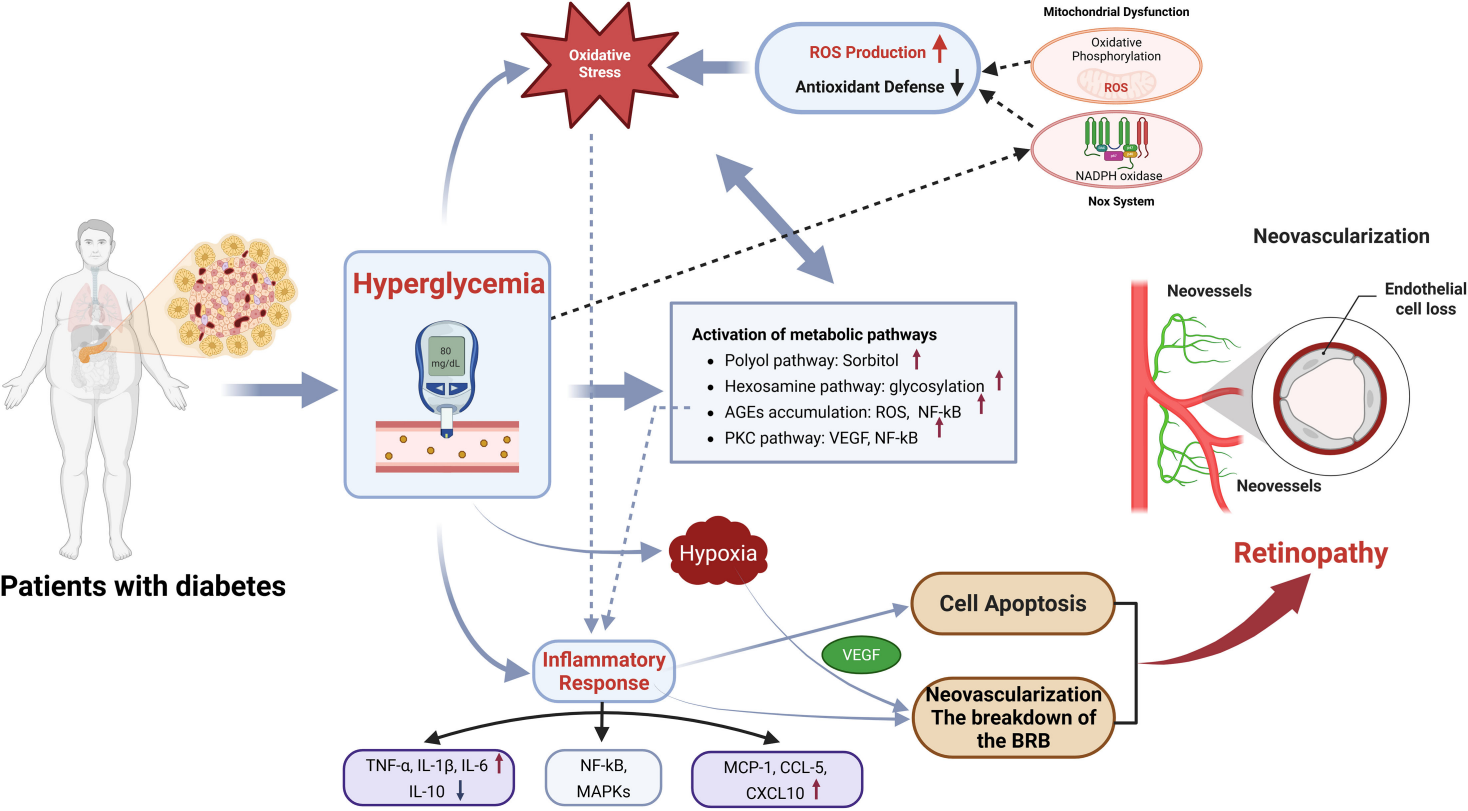
Mechanistic pathways linking hyperglycemia to DR. Chronic hyperglycemia activates multiple metabolic pathways, increases oxidative stress, and induces mitochondrial dysfunction, leading to excessive ROS production. These changes trigger inflammatory signaling (NF-κB, MAPKs), cytokine release, and hypoxia, collectively driving retinal cell apoptosis, BRB breakdown, and VEGF-mediated neovascularization. These processes converge to promote the onset and progression of DR and represent potential targets for exerkine-based therapeutic intervention.

DR is a multifactorial disease resulting from chronic hyperglycemia associated with diabetes. Traditionally, DR was viewed primarily as a vascular condition. However, recent research has shown that retinal nerve cell dysfunction occurs before retinal vasculopathy, leading to the current understanding of DR as a neurovascular disease (34–36). The pathological mechanisms underlying DR are complex and involve multiple interrelated processes, including metabolic, inflammatory, and neurodegenerative pathways. These mechanisms contribute collectively to the development and progression of DR, highlighting the multifactorial nature of the disease’s pathophysiology. Importantly, these processes do not act in isolation: hyperglycemia triggers metabolic disturbances that drive oxidative stress, which in turn activates inflammatory cascades. Inflammation further exacerbates oxidative injury and vascular leakage, establishing a self-perpetuating cycle that accelerates DR progression (37–39). Clarifying these mechanistic links helps explain why DR advances despite glycemic control and underscores the need for integrated therapeutic strategies (**Figure 1**).

### Hyperglycemia-induced damage

In the retina, neurons, glial cells, and vascular cells form an anatomically and functionally interdependent neurovascular unit (NVU), which is essential for maintaining tissue homeostasis and visual function (40–42). Early dysfunction of the NVU is now recognized as a central pathological event in the initiation of DR, as chronic hyperglycemia perturbs all cellular components within this unit (43, 44). Hyperglycemia activates multiple metabolic pathways, including the polyol and hexosamine pathways, the protein kinase C (PKC) pathway, and the formation of advanced glycation end products (AGEs), that collectively drive DR progression (37, 45). Excess glucose is reduced to sorbitol by aldose reductase, increasing intracellular sorbitol and Nicotinamide Adenine Dinucleotide (NADH) levels, inducing osmotic stress, and impairing retinal cell function (37, 46, 47). Concurrently, accelerated AGE accumulation leads to activation of the receptor for AGEs (RAGE) on endothelial cells and pericytes, amplifying oxidative stress and Nuclear Factor-κB (NF-κB)-mediated inflammatory signaling, thereby contributing to Blood-Retinal Barrier (BRB) disruption and pathological angiogenesis (37, 48, 49). Hyperglycemia-induced activation of the hexosamine pathway further dysregulates protein glycosylation and endothelial homeostasis (50, 51), while PKC, particularly the β-isoform, promotes vascular leakage, basement membrane thickening, and VEGF-driven neovascularization through NF-κB and VEGF upregulation (52–54). Together, these mechanisms underscore the therapeutic potential of targeting hyperglycemia-induced NVU damage as an early strategy for DR intervention. Moreover, interindividual variability in susceptibility to retinal injury suggests a genetic component, as polymorphisms in genes regulating oxidative stress, inflammatory signaling (e.g., NF-κB, VEGF), and metabolic enzymes such as aldose reductase may influence the onset and severity of DR (37, 55–57).

### Oxidative stress

Hyperglycemia induces pronounced retinal oxidative stress, leading to excessive accumulation of reactive oxygen species (ROS) that damage lipids, proteins, and DNA, thereby impairing cellular function and promoting cell death, key events in the onset and progression of DR (37, 58, 59). Mitochondrial oxidative phosphorylation and the NADPH oxidase (Nox) system constitute the two major enzymatic sources of ROS under hyperglycemic conditions and are central to the oxidative injury driving DR development (60, 61). Although endogenous antioxidant systems normally maintain redox balance, chronic hyperglycemia disrupts this equilibrium and results in sustained ROS overload. In particular, high glucose activates Nox enzymes, which transfer electrons from NADPH to oxygen to generate superoxide that is further converted to secondary ROS such as hydrogen peroxide (62–65). These Nox-derived oxidants, especially within retinal and vascular endothelial cells, exacerbate endothelial dysfunction and accelerate BRB breakdown, hallmark microvascular abnormalities leading to microaneurysms, hemorrhages, and neovascularization in DR (66). Importantly, mitochondrial and Nox-generated ROS act synergistically, each amplifying the other’s activity and creating a self-propagating cycle of oxidative stress that further intensifies retinal injury.

### Inflammation

Chronic hyperglycemia is a central driver of inflammation in DR, where sustained high glucose induces oxidative stress and accelerates the formation of advanced glycation end-products (AGEs), thereby initiating and perpetuating inflammatory cascades (67, 68). This response includes the upregulation of key pro-inflammatory cytokines, such as TNF-α, IL-1β, and IL-6, which disrupt the BRB, increase vascular permeability, and promote fluid leakage that leads to retinal edema (69–73). Concurrently, chemokines including CCL2 (MCP-1), CCL5, and CXCL10 are markedly elevated in the vitreous and serum of DR patients, facilitating leukocyte activation and recruitment; combined with inflammation driven by inducible nitric oxide synthase (iNOS) and cyclooxygenase-2 (COX-2), these mediators contribute to leukostasis, neutrophil infiltration, and microvascular injury (74, 75). The resulting capillary occlusion and ischemia further aggravate retinal damage while stimulating VEGF overexpression, which drives pathological neovascularization and increases the risk of vitreous hemorrhage and tractional retinal detachment in advanced DR (74). In parallel, hyperglycemia activates retinal microglia, promoting their release of additional cytokines and chemokines and thereby sustaining neuroinflammation, neuronal dysfunction, and cell death (76–79). At the molecular level, hyperglycemia activates multiple pro-inflammatory signaling pathways, including NF-κB (69, 80, 81) mitogen-activated protein kinase (MAPK) cascades (81, 82), and protein kinase C (PKC), particularly the PKC-β isoform, all of which amplify inflammatory mediator expression and adhesion molecule production (37, 81).

### Vascular permeability and neovascularization

Vascular permeability and neovascularization are central pathological processes driving the onset and progression of DR. Chronic hyperglycemia induces biochemical alterations in retinal microvasculature that compromise the integrity of the blood–retinal barrier, resulting in increased vascular permeability and the extravasation of fluid and proteins into surrounding tissues, which ultimately causes retinal swelling and visual distortion (42, 83–85). A major clinical manifestation of this breakdown is diabetic macular edema, a leading cause of vision loss. As prolonged hyperglycemia impairs retinal blood flow, tissue hypoxia develops, triggering the production of pro-angiogenic factors, most notably VEGF, that promote neovascularization (86). However, these newly formed vessels are structurally abnormal and highly fragile, making them prone to recurrent leakage and hemorrhage, which not only worsens edema but also facilitates fibrotic scar formation (87, 88). The resulting fibrovascular proliferation can exert traction on the retina, increasing the risk of tractional retinal detachment, a severe and vision-threatening complication that underscores the pathological role of aberrant neovascularization in advanced DR.

### Neurodegeneration

Recent evidence demonstrates that DR is not solely a vascular disorder but also features prominent neurodegenerative alterations that may precede the classical microvascular lesions traditionally associated with the disease (75, 87). Sustained hyperglycemia induces early retinal neurodegeneration, manifested as the loss of retinal ganglion cells, photoreceptors, and other neuronal populations (42), through mechanisms including oxidative stress, chronic low-grade inflammation, glutamate-mediated excitotoxicity, and mitochondrial dysfunction, all of which converge to promote neuronal apoptosis (89, 90). These pathological processes impair neurotrophic support, disrupt synaptic activity, and alter neurotransmitter release, resulting in functional deficits that can arise even before clinical signs of DR are detectable. As the disease progresses, neurodegeneration and vascular pathology interact synergistically, forming a vicious cycle wherein neuronal injury exacerbates microvascular compromise, while vascular insufficiency, particularly blood–retinal barrier breakdown, permits the leakage of toxic blood components that further amplify neuronal damage (13, 91). Importantly, neurodegeneration contributes independently to retinal dysfunction: patients may exhibit reduced contrast sensitivity, impaired dark adaptation, and abnormalities in visual processing prior to ophthalmoscopic evidence of vascular lesions (92–94). Consistent with this, electrophysiological assessments such as multifocal electroretinography have revealed delayed implicit times and diminished response amplitudes in diabetic eyes without visible retinopathy (95, 96), underscoring that neurodegenerative changes constitute an early and functionally significant component of DR pathogenesis.

### Endothelial dysfunction

Chronic hyperglycemia in diabetes drives excessive oxidative stress, leading to the overproduction of ROS that injure endothelial cells and diminish the bioavailability of nitric oxide (NO), a key vasodilator (97–100). Reduced NO signaling promotes vasoconstriction, platelet aggregation, and leukocyte adhesion, collectively accelerating retinal microvascular damage (101). In parallel, hyperglycemia-induced oxidative stress activates multiple pathological signaling cascades, including PKC, which further disrupt endothelial function and precipitate abnormal blood flow, vascular leakage, and neovascularization, hallmarks of DR (102–104). Moreover, diabetes-associated inflammation, characterized by elevated TNF-α, IL-6, and VEGF, amplifies endothelial activation, enhances leukocyte–endothelial interactions, and compromises the BRB (105, 106). The accumulation of AGEs and their interaction with RAGE receptors further intensify oxidative and inflammatory responses, thereby exacerbating endothelial dysfunction and promoting DR progression (107–109). Collectively, these interrelated mechanisms underscore DR as a systemic, multifactorial complication of chronic hyperglycemia, explaining why single-target interventions often yield limited benefit and highlighting the need for comprehensive, multi-dimensional therapeutic strategies.

## Overview of exercise and exerkines in DR

Regular physical activity is widely recognized for its numerous benefits, particularly in managing DR (110). Exercise improves insulin sensitivity and lowers blood glucose levels, which are essential for controlling diabetes (111, 112) and, consequently, the progression of DR (110, 113). Additionally, studies indicate that moderate exercise can slow the progression of DR by mitigating oxidative stress and inflammation, the primary factors driving retinal damage (110, 114, 115). Moreover, improved microcirculation and enhanced cardiovascular health, resulting from regular exercise, further contribute to protecting the retina by ensuring a better oxygen and nutrient supply to retinal tissues (116–118). In clinical practice, low-to-moderate intensity aerobic activities such as brisk walking, cycling, or swimming for at least 150 minutes per week are generally recommended for individuals with DR, as they can effectively improve glycemic control and vascular health while minimizing risks (119, 120). Resistance training using light to moderate loads can also be incorporated to enhance overall metabolic fitness (119–121). However, high-intensity or impact exercises that sharply increase intraocular pressure, such as heavy weightlifting or certain high-impact sports, should be avoided in individuals with advanced DR to reduce the risk of retinal detachment or vitreous hemorrhage (122, 123). Tailoring the exercise program to the patient’s stage of retinopathy and cardiovascular status is therefore essential for both safety and efficacy.

A growing body of evidence suggests that many of the systemic benefits of exercise on DR are mediated through exerkines, a diverse group of bioactive molecules released into circulation in response to physical activity (32, 124). These include proteins, peptides, cytokines, microRNAs, and metabolites that act as endocrine signals linking contracting muscles and other organs to distant tissues. Exerkines are thought to serve as a key molecular bridge between physical activity and retinal health, orchestrating multiple protective processes in the retina. Their anti-inflammatory and antioxidant properties help reduce inflammation and oxidative stress, two major contributors to retinal damage (125–127). Exerkines also regulate angiogenesis, promoting healthy blood vessel growth while preventing the abnormal vascularization associated with PDR (128, 129). Additionally, they exert neuroprotective effects, safeguarding retinal cells from apoptosis (130, 131), and improve glucose metabolism and insulin sensitivity, both critical for effective glycemic control (132, 133).

While many exerkines exert beneficial effects, it is important to acknowledge that some may have context-dependent or even detrimental roles, particularly in pathological states. For instance, certain pro-angiogenic or pro-inflammatory exerkines could theoretically exacerbate neovascularization or inflammation in susceptible individuals (32, 134, 135). Therefore, understanding the specific profile and balance of exerkines induced by different exercise modalities is crucial for translating these findings into targeted therapies.

## Therapeutic potential of exerkines in DR

**Figure 2:**
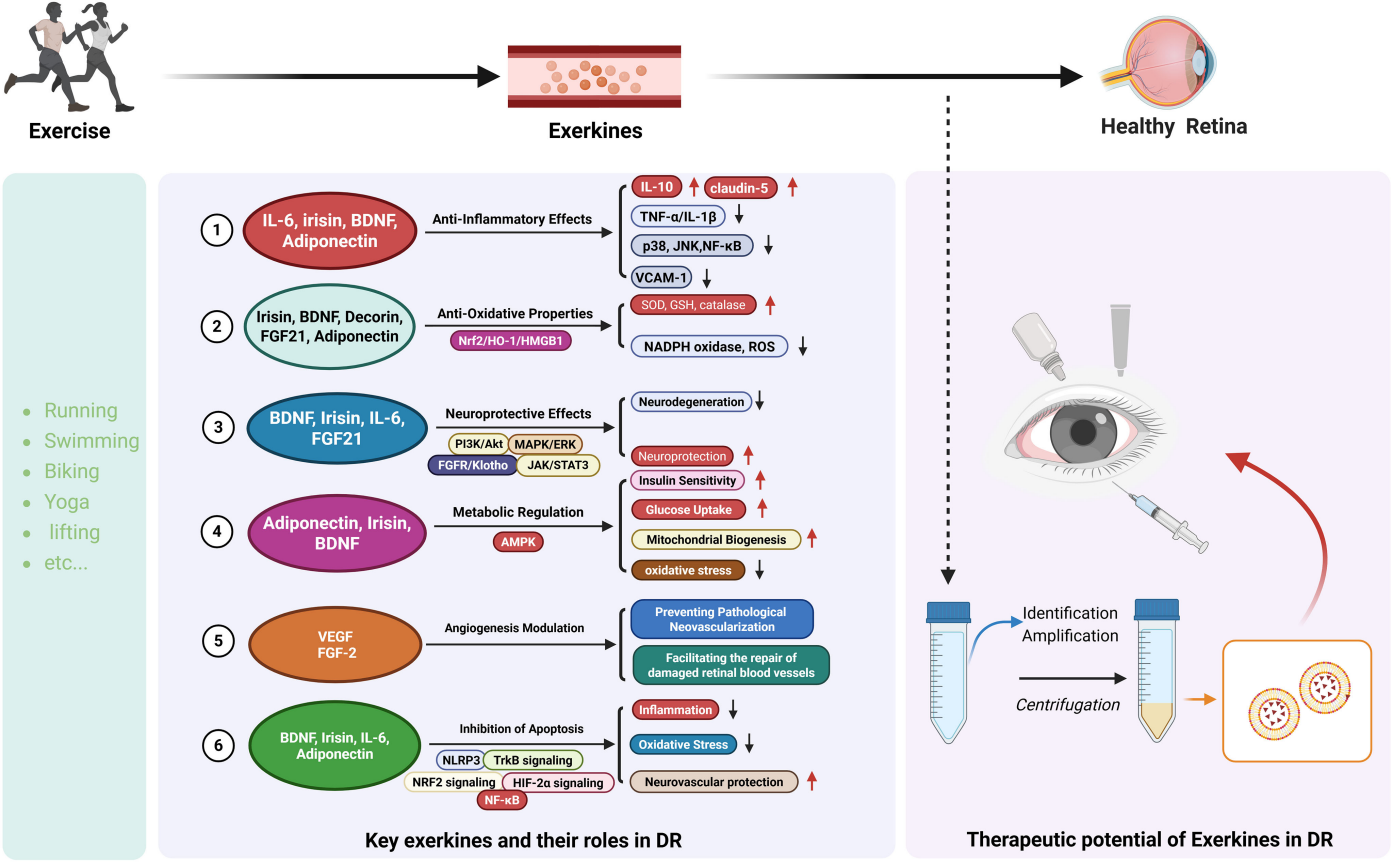
Key exerkines induced by exercise and their therapeutic roles in DR. Exercise stimulates the release of circulating exerkines, including IL-6, irisin, BDNF, adiponectin, FGF21, and decorin, which exert diverse protective effects on the diabetic retina. These exerkines modulate inflammation, oxidative stress, neurodegeneration, metabolism, angiogenesis, and apoptosis through pathways such as PI3K/Akt, MAPK/ERK, AMPK, Nrf2/HO-1, JAK/STAT3, and NF-κB. Collectively, they enhance mitochondrial function, glucose uptake, antioxidant defenses, insulin sensitivity, and vascular stability while suppressing ROS generation, inflammatory cytokines, and pathological neovascularization. The right panel illustrates the therapeutic potential of exerkines, highlighting their identification, amplification, and prospective delivery for retinal protection in DR.

Accumulating evidence highlights exerkines as potential therapeutic mediators in DR. These molecules exert multifaceted protective effects on retinal tissue by modulating inflammation, oxidative stress, neuronal survival, metabolism, angiogenesis, and apoptosis, as illustrated in **Figure 2**. While most insights derive from broader metabolic or neurodegenerative disease contexts, the mechanisms provide a compelling rationale for exploring exerkines in DR management.

### Anti-inflammatory effects of exerkines in DR

Exerkines exert broad anti-inflammatory effects that may mitigate retinal inflammation in DR. Key mediators released during physical activity, including IL-6, irisin, Brain-Derived Neurotrophic Factor (BDNF), and adiponectin, suppress pro-inflammatory cytokines, inhibit major inflammatory pathways such as NF-κB, and preserve retinal homeostasis (**Table 1**). Importantly, these effects are cell-type specific and context dependent, varying across disease stage and retinal microenvironment.

**Table 1:** The role of exerkines in retinal cells.

Retinal cell type	Key exerkines	Major receptors	Downstream pathways modulated	Functional outcome	References
Microglia	BDNF, irisin	TrkB, integrin/AMPK-related	↓ p38/JNK/NF-κB	Reduced activation, ↓ IL-6/TNF-α	(136–143)
Müller glia	IL-6, adiponectin	IL-6R/gp130, AdipoR1/R2	Context-dependent NF-κB, ↓ oxidative stress	Cytoprotection, barrier support	(134, 144–152)
Endothelial cells	IL-10, adiponectin	IL-10R, AdipoR1/R2	↓ NF-κB, ↓ VCAM-1, ↑ claudin-5	Preserved BRB integrity	(150–154)
Neurons	BDNF	TrkB	Pro-survival, anti-inflammatory signaling	Neuroprotection	(136–140, 155)

“↓” indicates a decrease, downregulation, or inhibition; “↑” indicates an increase, upregulation, or activation (relative to diabetic pathological baseline as described in context).

### Microglia

Microglia are central drivers of neuroinflammation in DR and represent a major target of exercise-induced exerkines. Exercise-stimulated BDNF suppresses microglial activation and limits IL-6 and TNF-α production through inhibition of p38, c-Jun N-terminal kinase (JNK), and NF-κB signaling, thereby fostering a neuroprotective retinal milieu (156–162). Irisin similarly dampens microglial inflammatory responses by repressing NF-κB activation and reducing pro-inflammatory cytokine release (163, 164).

### Müller glia

Müller glial cells function as metabolic and immunoregulatory hubs within the retina. Exerkines, particularly IL-6 and adiponectin, exert nuanced effects on these cells. During exercise, skeletal muscle-derived IL-6 acts predominantly through classical IL-6 signaling, conferring cytoprotection and attenuating glucose-induced oxidative stress and inflammation (125, 136–138). A recent review highlights the role of IL-6, demonstrating that exercise-induced IL-6 exerts potent anti-inflammatory, antioxidant, and stress-alleviating effects (139). In contrast, IL-6 trans-signaling, which may become more prominent in advanced or proliferative DR, has been associated with enhanced inflammatory responses and gliosis, highlighting a disease stage-dependent functional switch (140, 141). Adiponectin further supports Müller cell function by suppressing NF-κB/TNF signaling and reducing oxidative stress (142–144).

### Retinal endothelial cells

Inflammation-driven endothelial dysfunction underlies BRB breakdown in DR. Several exerkines directly protect retinal endothelial cells by enhancing tight junction integrity and reducing leukocyte adhesion. IL-10, induced downstream of exercise-associated IL-6 signaling, limits inflammatory macrophage accumulation and preserves BRB function (145). Adiponectin signaling suppresses vascular cell adhesion molecule-1 (VCAM-1) expression and maintains claudin-5 expression, whereas adiponectin deficiency exacerbates vascular leakage and endothelial inflammation (142–144, 146).

### Neurons

Although less extensively studied, retinal neurons also benefit from exerkine signaling. BDNF, a neurotrophin strongly induced by exercise, supports neuronal survival and synaptic integrity while counteracting inflammation-associated neurodegeneration, both directly and indirectly through suppression of microglial activation (147, 156–160).

### Context-dependent and dual roles of exerkines

While many exerkines exert protective anti-inflammatory effects in early or moderate DR, their roles may shift as disease progresses. IL-6 exemplifies this duality: transient, exercise-induced IL-6 signaling suppresses TNF-α and IL-1β and promotes IL-10 production, whereas sustained IL-6 elevation, particularly via trans-signaling, may amplify inflammation, angiogenesis, and fibrosis in proliferative DR (125, 136–138). Similarly, apelin may enhance endothelial survival and metabolic adaptation in early DR but promote pathological angiogenesis and vascular remodeling at later stages, as demonstrated in animal models (148–150).

### Anti-oxidative properties

Exercise and its released exerkines play a pivotal role in counteracting oxidative stress in DR through multiple convergent mechanisms. A key effect is the enhancement of endogenous antioxidant defenses, including increased production of superoxide dismutase (SOD) and glutathione (GSH), which strengthen cellular resistance to ROS. Mild ROS generated during exercise can activate protective signaling cascades, promoting the expression of antioxidant enzymes and other cytoprotective systems (114). Concurrently, exercise suppresses NADPH oxidase, one of the primary enzymatic sources of ROS, thereby further reducing oxidative burden (151). These antioxidant responses have clear relevance to DR, as elevated SOD, catalase, and glutathione peroxidase levels correlate with reduced oxidative injury in retinal tissues, and inhibition of NADPH oxidase effectively prevents pathological ROS overproduction (61, 152, 153). Moreover, exerkines such as irisin activate the Nrf2/HO-1/HMGB1 pathway (154, 155), a central regulator of the antioxidant response, while others including decorin, BDNF, adiponectin, and FGF21 may also contribute to mitigating oxidative stress (165–168).

Beyond direct antioxidant actions, exerkines modulate inflammatory signaling, a process closely intertwined with oxidative stress in DR, by suppressing pro-inflammatory cytokines and enhancing anti-inflammatory mediators (124, 169). They also improve mitochondrial function by promoting mitochondrial biogenesis and optimizing energy metabolism, thereby limiting ROS generation at its source (170–172). Collectively, these coordinated effects suggest that exerkines may provide a more robust and physiologically integrated defense against oxidative stress–driven retinal damage. However, empirical evidence specific to DR remains limited, underscoring the need for further investigation. Notably, conventional antioxidant therapies, including vitamins C and E or pharmacological Nrf2 activators, have shown disappointing clinical efficacy due to poor bioavailability, inadequate retinal delivery, and inability to neutralize sustained oxidative insults (37, 173, 174). In contrast, exerkine-mediated mechanisms are endogenous, dynamic, and cell-type specific; by simultaneously enhancing antioxidant capacity, inhibiting ROS-generating pathways, and improving mitochondrial resilience (28, 175–177), exerkines may overcome the inherent limitations of single-agent antioxidant strategies and offer a more durable therapeutic approach for DR.

### Neuroprotective effects

Exerkines exert potent neuroprotective effects that are essential for maintaining retinal neuronal integrity in DR (130, 178). Exercise-induced factors such as irisin, IL-6, BDNF, and FGF21 collectively modulate inflammation, attenuate oxidative stress, and enhance neurotrophic and mitochondrial support, thereby preventing hyperglycemia-induced neurodegeneration. Irisin and IL-6 reduce pro-inflammatory cytokine expression, limiting chronic inflammation that drives retinal injury (125, 179), whereas BDNF and irisin strengthen endogenous antioxidant defenses to prevent ROS-mediated neuronal damage (180–182). Neurotrophic support, particularly through BDNF, further promotes neuronal survival and regeneration, offering protection not only in DR but also in neurodegenerative diseases such as glaucoma (52, 130, 183). These functional benefits are mediated through convergent intracellular signaling cascades: BDNF activates tropomyosin receptor kinase B (TrkB)-dependent Phosphatidylinositol 3-kinase/Protein kinase B (PI3K/Akt) and Mitogen-activated protein kinase/Extracellular signal-regulated kinase (MAPK/ERK) pathways to enhance survival and synaptic plasticity, reducing apoptosis and preserving retinal structure in hyperglycemic conditions (34, 162, 183); irisin similarly stimulates PI3K/Akt and MAPK signaling to improve mitochondrial function and suppress apoptosis (184, 185); IL-6 engages the gp130/JAK/STAT3 axis to regulate transcriptional programs governing neuronal survival, inflammatory resolution, and retinal repair (125, 186–188); and FGF21 activates extracellular signal-regulated kinase 1/2 (ERK1/2) and AMP-activated protein kinase (AMPK) pathways downstream of Fibroblast growth factor receptor/Klotho (FGFR/Klotho), thereby supporting mitochondrial homeostasis and antioxidative responses, consistent with its protective effects in diabetic retinal models (58, 189). Together, these interconnected mechanisms underscore how exerkines form an integrated protective network that preserves retinal neurons under diabetic conditions.

### Metabolic regulation

Exercise-induced exerkine production is tightly regulated by key metabolic processes, including insulin sensitivity, AMPK activation, and mitochondrial biogenesis, that collectively influence the development of DR (190–193). Improved insulin sensitivity following exercise modulates the secretion of several exerkines, particularly adiponectin, thereby enhancing systemic glucose control and reducing hyperglycemia, a primary driver of retinal vascular damage (192, 194–196). Concurrently, exercise activates AMPK, a central regulator of glucose uptake, fatty acid oxidation, and the expression of cytoprotective exerkines such as irisin (197, 198) and BDNF (199), which may confer direct retinal protection. In parallel, mitochondrial biogenesis induced by exercise helps maintain metabolic homeostasis and attenuate oxidative stress in retinal cells, further contributing to DR prevention (170, 200–202). Beyond these local effects, systemically released myokines such as irisin (203) and adipokines (204) during exercise also enhance insulin sensitivity and glucose metabolism (203), indirectly supporting retinal health by limiting hyperglycemia-mediated vascular injury. Together, these coordinated metabolic adaptations underscore the multifaceted protective role of exercise-regulated exerkines in preserving retinal integrity under diabetic conditions.

### Angiogenesis modulation

Angiogenesis, the formation of new blood vessels, is a critical factor in the progression of DR, particularly in its proliferative stage (205). Exercise has a significant impact on modulating this process (206), primarily through the influence of exerkines like VEGF (193) and fibroblast growth factor-2 (FGF-2) (207), which are crucial in angiogenesis. Exercise-induced changes in VEGF levels can help normalize the angiogenic process by facilitating the repair of damaged retinal blood vessels and preventing pathological neovascularization (208–210). Although VEGF is typically upregulated in DR, most studies investigating this mechanism have not specifically focused on the role of exerkines in regulating angiogenesis within DR models, highlighting the need for further research to validate these findings.

### Inhibition of apoptosis

Exercise-induced exerkines exert potent anti-apoptotic effects in retinal cells through coordinated antioxidant, anti-inflammatory, and neurovascular protective mechanisms. These factors enhance intrinsic antioxidant defenses, primarily by upregulating enzymes such as superoxide dismutase and catalase, which neutralize ROS, restore redox homeostasis, and prevent oxidative-stress-driven apoptotic signaling (211–213). Concurrently, exerkines suppress chronic inflammation by downregulating pro-inflammatory cytokines including TNF-α and IL-6, thereby limiting inflammatory cascades that promote apoptosis in retinal degenerative conditions (192, 214). Key mediators such as irisin, BDNF, IL-6, adiponectin, FGF21, and apelin each contribute distinct yet complementary protective actions: irisin inhibits NLRP3 inflammasome activation, attenuates oxidative stress, and reduces pyroptotic cell death (215); BDNF engages TrkB signaling to preserve retinal ganglion cells and maintain neuronal function (52); IL-6 exerts anti-inflammatory and metabolism-regulating effects that may protect retinal cells (136); and adiponectin reduces retinal apoptosis through anti-inflammatory and insulin-sensitizing actions (216). In DR, BDNF further supports neural preservation by activating TrkB signaling and preventing early neuronal decline (217), while FGF21 enhances NRF2-mediated antioxidant defenses and suppresses IL-1β and TNF-α to maintain neurovascular integrity and photoreceptor survival (58, 218). Irisin additionally attenuates pathological neovascularization by downregulating VEGFA and inhibiting NF-κB and HIF-2α signaling (219, 220), whereas apelin strengthens vascular stability by upregulating tight junction proteins (ZO-1, occludin) and protecting pericytes, thereby limiting vascular leakage (149, 221). In parallel, adiponectin reduces TNF-α levels, decreases leukocyte adhesion, and suppresses aberrant neovascularization (216, 222). Collectively, these exerkines form an integrated protective network that combines neuroprotection with antioxidative, anti-inflammatory, and vascular-stabilizing functions, highlighting exercise as a promising systemic intervention to mitigate retinal degeneration and slow the progression of DR.

## Exercise modality: specific exerkine signatures relevant to DR

Exercise should not be conceptualized as a single, uniform “dose.” Distinct modalities, including aerobic endurance exercise, high-intensity interval training (HIIT), and resistance training, elicit partially overlapping yet clearly differentiated exerkine signatures, encompassing myokines, metabolites, hepatokines, and extracellular vesicle (EV) cargo. This distinction is particularly relevant to DR, a multifactorial disease driven by intertwined processes such as BRB breakdown, pathological angiogenesis, chronic inflammation, oxidative stress, and early retinal neurodegeneration (28, 110, 176). Accordingly, a modality-oriented framework is required to identify exercise-responsive signals with the strongest mechanistic relevance to DR, while acknowledging that evidence remains incomplete for several pathways (**Table 2**).

**Table 2:** Exercise modalities, their effects on regulating exerkines, and clinical significance in DR.

Exercise modality	Putative “signature” exerkines	DR-relevant pathway modules most likely impacted	Evidence level for DR relevance	References
MICT	BDNF/TrkB axis, lactate (as a coupling metabolite), acute myokine IL-6 pulse (context-dependent).	Retinal neuroprotection, anti-apoptosis; potential indirect anti-inflammatory remodeling; systemic cardiometabolic improvements reduce upstream DR drivers.	Strong preclinical retina evidence that exercise protects diabetic retina and involves TrkB signaling (blocking TrkB blunts protection).	(222–224)
HIIT	Higher-amplitude lactate pulses; candidate amplification of BDNF-linked programs.	Potentially stronger neurotrophic/metabolic signaling, but also larger acute stress responses (oxidative/inflammatory) in some patients, heterogeneous net retinal effect.	Mechanistic plausibility extrapolated from lactate/BDNF coupling and exercise-TrkB retinal protection literature; direct DR endpoint trials+exerkine profiling remain limited.	(224)
RT	Irisin; other load-responsive myokines	Anti-angiogenic / anti-inflammatory signaling (hypoxia-HIF/VEGF, NF-κB), cytoprotection; strong systemic insulin-sensitizing effects.	Irisin attenuates pathological retinal neovascularization and reduces inflammation/apoptosis in oxygen-induced retinopathy (OIR), a mechanistically relevant proliferative angiogenesis model.	(225–228)
Combined training	BDNF/TrkB + irisin + improved systemic anti-inflammatory milieu.	Simultaneous engagement of neuroprotection+vascular stabilization, plus upstream metabolic risk reduction.	Inference-based synthesis from modality-specific mechanistic studies above (BDNF/TrkB retinal protection; irisin anti-neovascular signals).	(149, 225, 229)

### MICT

Moderate-intensity continuous aerobic training (MICT) generates a characteristic “neurovascular-stabilizing” exerkine profile. Repeated bouts of sustained muscle contraction induce transient endocrine pulses rather than chronic elevations, favoring anti-inflammatory and neurotrophic signaling while simultaneously improving glycemic control and blood pressure, two major upstream determinants of DR progression (28, 110, 176). Preclinical studies and human genetic epidemiology further support a link between habitual aerobic exercise and retinal neuroprotection. Among candidate mediators, BDNF is particularly compelling. In diabetic rodents, exercise preserves visual and retinal function, whereas pharmacologic blockade of TrkB signaling abolishes this benefit, supporting a causal BDNF-TrkB axis in retinal resilience (217, 225, 230). Clinically, reduced retinal BDNF expression has been associated with early diabetic retinal apoptosis and neurodegeneration, suggesting that restoration of BDNF signaling may modify disease trajectories upstream of overt microvascular pathology (162, 231). Because MICT consistently elevates circulating BDNF, it represents a rational foundational prescription when neuroprotection is a primary therapeutic goal (217, 232).

Additionally, IL-6 illustrates the context-dependent nature of exercise-induced exerkines. During endurance exercise, skeletal muscle-derived IL-6 rises acutely and can exert systemic anti-inflammatory effects, including suppression of TNF-α and IL-1β within the exercise milieu (22, 233). In contrast, intraocular and retinal IL-6 signaling has been implicated in BRB disruption, vascular leakage, and inflammatory activation, particularly via IL-6 trans-signaling pathways that impair endothelial barrier integrity (71, 223, 224). Importantly, emerging evidence suggests that exercise-induced lactate contributes to retinal protection and interacts with BDNF/TrkB-dependent signaling pathways (176, 225). Thus, aerobic modalities that repeatedly elicit modest lactate elevations, without provoking excessive metabolic or inflammatory stress, may reinforce neurotrophin-linked retinal resilience while minimizing adverse inflammatory signaling.

### HIIT

HIIT produces a distinct “high-amplitude pulse” exerkine signature characterized by short bursts near maximal oxygen uptake, leading to pronounced transient increases in lactate, catecholamines, and metabolic stress signals. In theory, stronger lactate-BDNF coupling could amplify neuroprotective pathways relevant to DR, consistent with experimental data linking lactate signaling to retinal BDNF-dependent protection (225). However, HIIT may also provoke greater acute oxidative and inflammatory responses, and individuals with DR frequently present with comorbid hypertension or cardiovascular disease. Consequently, the net retinal benefit of HIIT is likely to be highly context-dependent, shaped by individual tolerance, baseline glycemic control, and vascular risk. Given the current scarcity of DR-specific exerkine and clinical endpoint studies, HIIT should be considered a promising but still exploratory strategy requiring careful safety stratification and targeted validation.

### RT

Resistance training (RT) elicits a complementary “myokine-dominant and insulin-sensitizing” signature with distinct implications for vascular and inflammatory regulation. Resistance exercise is consistently associated with improvements in metabolic control, redox balance, and systemic inflammation in diabetes-related vascular disease (229, 234, 235). Irisin, a myokine preferentially induced by resistance and combined training, has been shown to attenuate pathological retinal neovascularization in oxygen-induced retinopathy models, a mechanistic analogue of proliferative DR (219). These findings support the concept that resistance-driven exerkines may preferentially target oxidative stress and angiogenic pathways, thereby complementing aerobic, neurotrophin-centered mechanisms in a multimodal exercise prescription.

### Endurance and combined training

Endurance and combined training modalities that improve hepatic lipid handling may therefore provide a mechanistically coherent route to both early neuroprotection and later vascular stabilization (162, 219, 231). Besides, exercise modulates EV release and circulating microRNA profiles, including endothelial-enriched miRNAs such as miR-126, which are responsive to training in metabolic disease contexts (236, 237). Although DR-specific causal evidence remains limited, miRNAs are recognized regulators of angiogenesis, inflammation, oxidative stress, and neurodegeneration in DR pathogenesis (226). Notably, aerobic training appears to elicit clearer endothelial and miR-126-related signals in diabetes cohorts, positioning it as a particularly suitable modality for EV-miRNA biomarker discovery in DR (236, 237).

### Evidence from preclinical and clinical studies

Regular physical activity reshapes the systemic secretome, increasing circulating levels of myokines, adipokines, hepatokines, and neurotrophins, collectively termed exerkines, that exert endocrine effects on distal organs, including the retina. Over the past decade, a growing body of *in vitro*, animal, and human data has begun to link these exercise-inducible factors to key pathogenic nodes in DR, including vascular leakage, neurodegeneration, inflammation, and oxidative stress.

### *In vitro* retinal cell models

#### Retinal endothelial cells

High-glucose and AGE models in retinal endothelial cells have been used to test the direct actions of candidate exerkines on barrier integrity, apoptosis and angiogenesis. In rhesus choroid-retinal endothelial RF/6A cells, adiponectin restored cell viability and suppressed high glucose-induced migration and tube formation, while shifting the Bax/Bcl-2 balance towards cell survival and reducing apoptosis (227). These effects were accompanied by inhibition of autophagy and activation of PI3K/Akt/mTOR signaling, indicating that adiponectin can normalize endothelial stress responses and pathological angiogenic behavior under diabetic conditions (227). Clusterin, a secreted chaperone upregulated by cellular stress and increasingly recognized as a putative exercise‐responsive factor, has been shown to protect human retinal microvascular endothelial cells (HRMECs) from VEGF- and AGE-induced hyperpermeability. Up to 20 µg/mL clusterin did not impair HRMEC viability, yet markedly reduced paracellular flux and restored tight junction proteins (ZO-1, occludin, claudin-5), thereby preventing blood-retinal barrier (BRB) breakdown in diabetic models (228, 238, 239). These findings directly address a central functional readout-barrier function, and link an exerkine-like factor to structural preservation of the retinal vasculature.

#### Retinal pigment epithelial cells

The RPE is highly susceptible to oxidative and metabolic stress, and several exerkine candidates modulate its survival. Clusterin protects human RPE (ARPE-19) cells from oxidative stress-induced apoptosis by activating PI3K/Akt signaling and inhibition of PI3K abolishes clusterin’s pro-survival effect (239, 240). Given that systemic clusterin levels are influenced by cardiometabolic status and physical training, this pathway offers a plausible mechanistic link between exercise, circulating clusterin, and preservation of RPE integrity in diabetes.

#### Neurons and neurotrophins

BDNF is one of the best-characterized exercise-inducible neurotrophins. In cultured retinal neurons exposed to hyperglycemia, exogenous BDNF preserved cell viability and reduced apoptosis via TrkB/ERK/MAPK signaling, implicating this axis in neuroprotection against glucose toxicity (162, 183, 241). Exercise is known to increase BDNF in the retina and serum (160, 162, 217, 225), and in rodent retinal injury models, exogenous irisin, a myokine that upregulates BDNF, attenuates neuroinflammation and ganglion cell death, supporting a broader “exerkine-BDNF” pathway in retinal neuroprotection (162, 219, 239).

#### ROS and inflammatory markers

**Figure 3:**
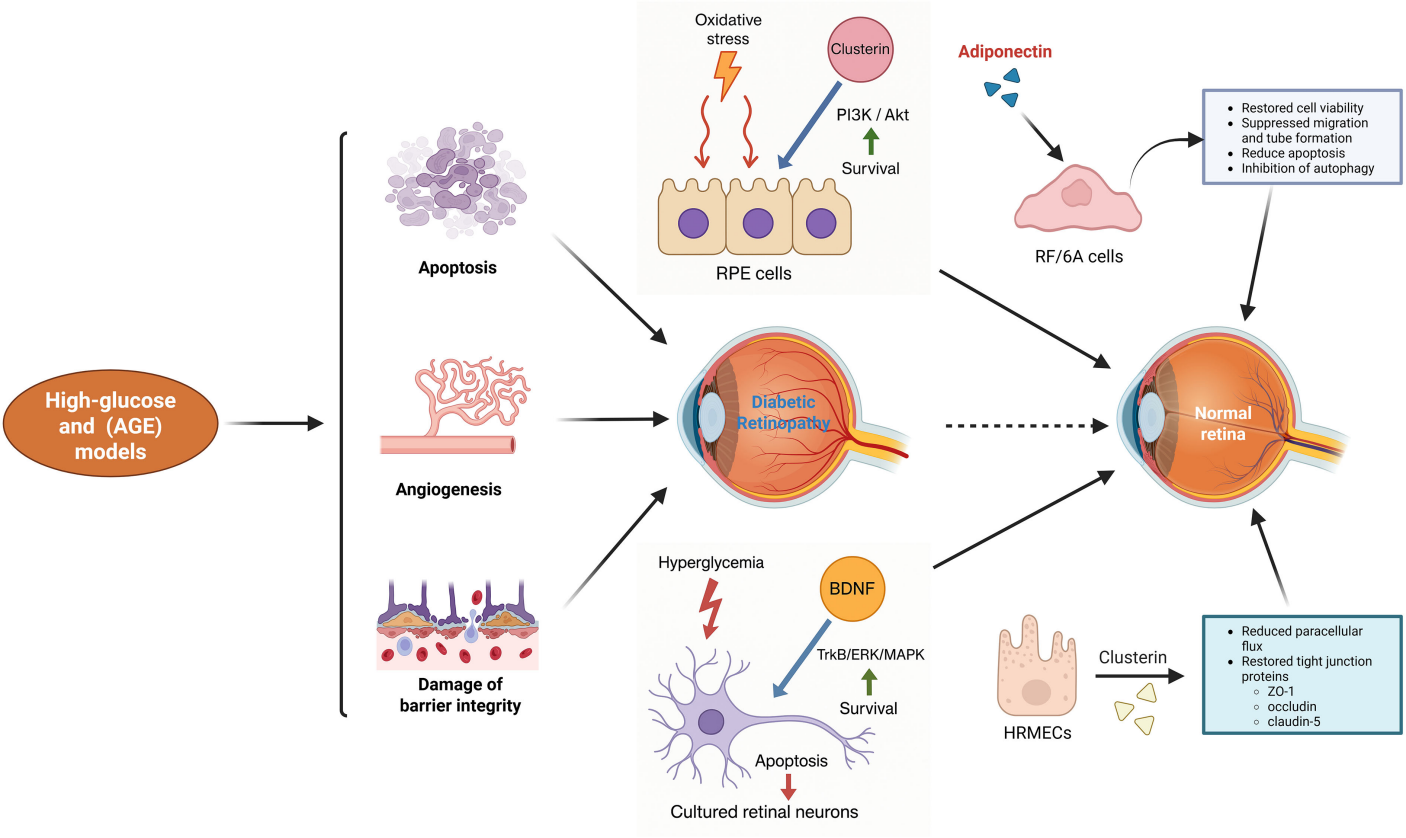
Protective effects of key exerkines on cellular injury mechanisms relevant to DR. High-glucose and AGE conditions induce apoptosis, pathological angiogenesis, and disruption of barrier integrity, contributing to DR. Exerkines such as clusterin, adiponectin, and BDNF counteract these damaging processes through complementary mechanisms. Clusterin enhances PI3K/Akt survival signaling in RPE cells and restores tight-junction proteins (ZO-1, occludin, claudin-5) in HRMECs, improving barrier function. Adiponectin preserves RF/6A cell viability, suppresses migration and tube formation, reduces apoptosis, and inhibits autophagy. BDNF activates TrkB/ERK/MAPK signaling in retinal neurons, promoting survival and reducing hyperglycemia-induced apoptosis. In short, these exerkines mitigate glucose-driven cellular injury and support restoration of a healthier retinal environment.

Across these *in vitro* systems, exerkine treatment typically converges on reduction of oxidative stress and inflammatory signaling: adiponectin diminishes high glucose-induced angiogenic and pro-apoptotic responses while normalizing autophagy (227); clusterin reduces oxidative-stress-induced apoptosis in RPE and retinal endothelial cells (228, 238); and BDNF-related signaling dampens stress kinase activation in retinal neurons (162, 183). Collectively, these *in vitro* data provide direct mechanistic evidence that exerkine pathways can modulate cell viability, apoptosis, barrier function, ROS generation and inflammatory mediators in retinal cells relevant to DR (**Figure 3**).

## Animal models of DR

**Figure 4:**
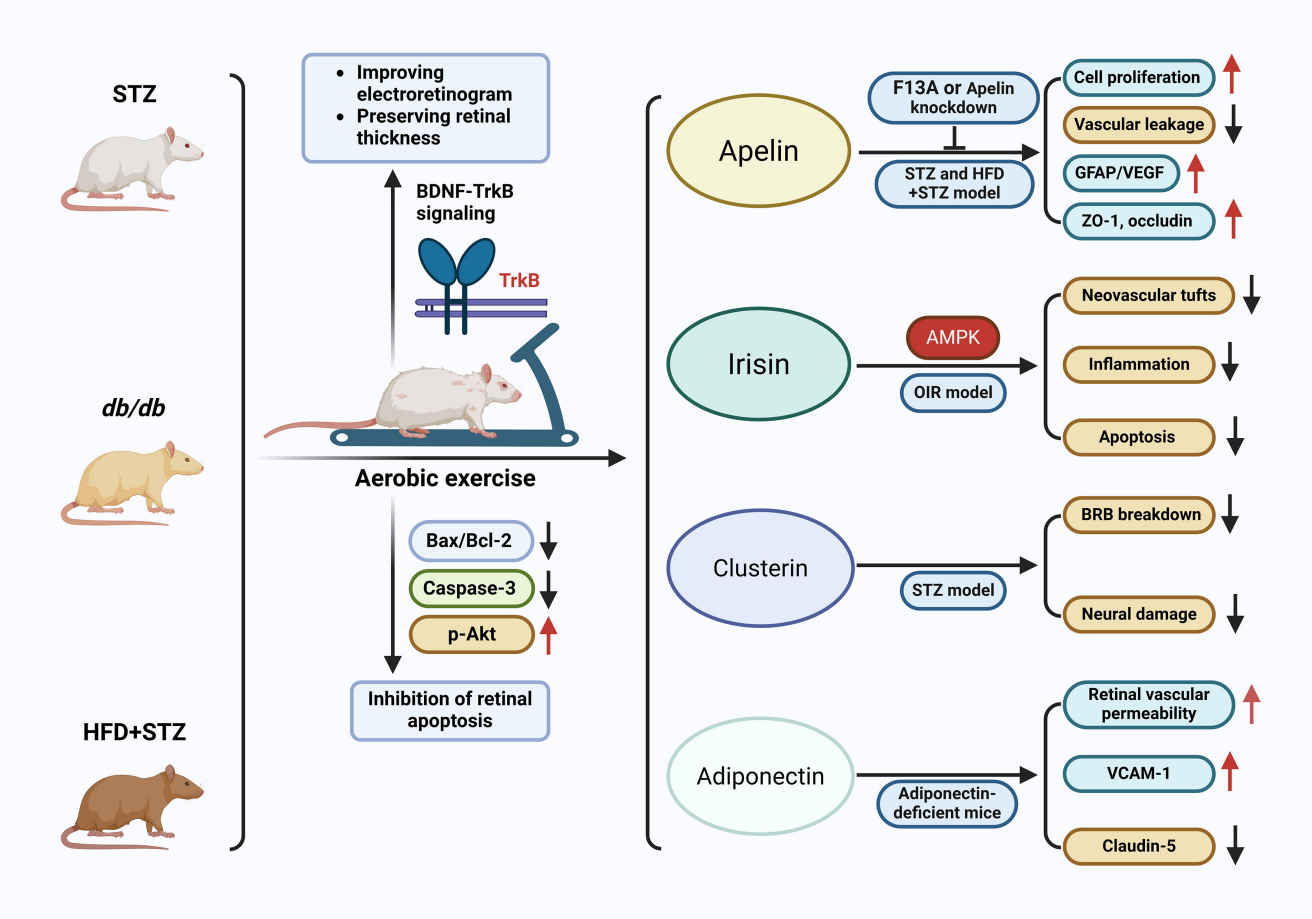
Exercise-induced exerkines and their protective actions in experimental models of DR. Aerobic exercise improves retinal function and structure in STZ-, db/db-, and HFD+STZ-induced diabetic mouse models, partly through activation of BDNF-TrkB signaling and inhibition of retinal apoptosis, as evidenced by reduced Bax/Bcl-2 ratio and caspase-3 expression, alongside increased p-Akt. Individual exerkines exert complementary therapeutic effects: Apelin suppresses vascular leakage and inflammation while restoring tight junction proteins; irisin, via AMPK activation, reduces neovascularization, inflammation, and apoptosis in OIR models; clusterin attenuates BRB breakdown and neural damage in STZ-induced injury; and adiponectin improves vascular integrity by decreasing VCAM-1 and enhancing claudin-5 expression. Together, these exerkines represent key mediators through which exercise confers retinal protection in DR.

Exercise training in STZ, db/db and HFD+STZ models. Multiple studies using streptozotocin (STZ)-induced type 1 diabetes, db/db mice and high-fat diet (HFD)+STZ type 2 diabetes models have shown that aerobic exercise preserves retinal structure and function (160, 176, 217, 225, 242). In STZ-diabetic rats, treadmill exercise reduced retinal apoptosis (TUNEL+cells, Bax/Bcl-2 ratio, caspase-3 activation) and restored p-Akt levels in the retina, indicating activation of survival signaling in the diabetic neurovascular unit (242). In an elegant series of experiments, STZ-diabetic rats subjected to moderate treadmill exercise exhibited improved electroretinogram (ERG) responses and preserved retinal thickness, while pharmacologic blockade of TrkB abolished these benefits (217). This firmly implicated BDNF-TrkB signaling in exercise-mediated retinal protection. Subsequent work and reviews have extended these observations to other models, showing that exercise preserves retinal ganglion cell survival, neurovascular coupling and synaptic integrity (160, 162, 176). Although many of these studies did not directly quantify exerkines, they provide a functional context in which exercise-induced molecules such as BDNF, irisin, adiponectin and clusterin are likely mediators, as shown in **Figure 4**.

### Apelin/APJ signaling in diabetic retinas

Apelin, a myokine/adipokine upregulated by exercise, has a complex role in DR. In STZ-diabetic rats, apelin expression increases in Müller cells and retinal vessels, and exogenous apelin promotes cell proliferation and GFAP/VEGF upregulation, while the apelin receptor antagonist F13A suppresses Müller cell proliferation and reduces gliotic and angiogenic markers, suggesting a pathogenic role in proliferative stages (243, 244). In contrast, a more recent study in HFD+STZ-induced type 2 diabetic mice used intravitreal lentiviral vectors to modulate apelin expression and reported that apelin overexpression (LV-Apelin^+^) preserved pericyte density, reduced vascular leakage, and upregulated tight junction proteins ZO-1 and occludin, whereas apelin knockdown aggravated pericyte loss and leakage (149). These findings suggest that apelin’s impact may be stage-dependent-protective for early microvascular integrity yet potentially pro-proliferative under chronic hypoxic conditions, highlighting the need for careful therapeutic targeting.

### Irisin and pathological neovascularization

In oxygen-induced retinopathy (OIR), a model of ischemic proliferative retinopathy, retinal irisin expression is downregulated, and intravitreal irisin supplementation reduces pathological neovascular tufts, inflammation and apoptosis while partially normalizing vascular density (219). Although OIR is not a diabetic model, it shares key angiogenic and inflammatory pathways with proliferative DR, supporting an anti-angiogenic, vasoprotective role for irisin that could be harnessed in diabetic settings. Rodent work in other organs indicates that irisin activates AMPK and reduces inflammasome activity under hyperglycemic stress, further strengthening its candidacy as a DR-targeted exerkine (219, 238).

### Clusterin and adiponectin in diabetic retina

In STZ-diabetic rats, retinal clusterin expression is upregulated, particularly around vascular lesions, and exogenous clusterin reduces BRB breakdown and neural damage, likely by stabilizing tight junctions and limiting apoptosis (238, 239, 245). Animal data also support a protective role for adiponectin: adiponectin-deficient mice exhibit exaggerated retinal vascular permeability, increased VCAM-1 and reduced claudin-5 under hyperglycemia, whereas restoration of adiponectin signaling normalizes endothelial barrier markers and reduces leakage (216, 246). These *in vivo* findings dovetail with *in vitro* RF/6A data (227), and collectively suggest that adiponectin and clusterin, both influenced by metabolic fitness, contribute to retinal vascular resilience in diabetes. Overall, data from STZ, db/db and HFD+STZ models demonstrate that (i) structured exercise training can mitigate DR-like changes at histologic, functional (ERG) and molecular levels, and (ii) direct manipulation of exerkine pathways (apelin, irisin, adiponectin, clusterin, BDNF) modulates pericyte survival, vascular leakage, neovascularization, apoptosis and inflammatory signaling in the diabetic or ischemic retina.

## Human observational evidence linking physical activity to DR

Across observational studies, higher habitual physical activity (PA) is consistently associated with lower DR prevalence and slower progression, although causality cannot be inferred. A large meta-analysis of more than 63,000 participants from 22 studies reported significantly reduced risks of any DR and vision-threatening DR among individuals with higher PA levels, with several analyses suggesting partial independence from glycemic control (21). Nevertheless, substantial heterogeneity exists due to differences in PA assessment (self-reported vs. objective), DR classification, and covariate adjustment. Prospective cohort studies provide more robust support. In patients with type 2 diabetes, higher PA thresholds were associated with a lower incidence of DR after adjustment for major confounders, including age, diabetes duration, HbA1c, blood pressure, and lipid profiles (247). In type 1 diabetes, higher leisure-time PA has been linked to a reduced risk of laser-treated severe DR. However, attenuation after adjustment for HbA1c and smoking suggests that improved metabolic control may partially mediate this association (248). Cross-sectional analyses consistently demonstrate that patients with advanced DR accumulate less total PA and exhibit greater sedentary behavior compared with those with mild DR or no retinopathy (249). While these findings support a relationship between habitual inactivity and microvascular disease burden, reverse causality, whereby visual impairment or systemic comorbidity limits activity, cannot be excluded. More recently, accelerometer-based studies have strengthened the evidence by objectively quantifying PA. In large cohorts of individuals with type 2 diabetes, higher levels of moderate-to-vigorous PA, particularly during daytime hours, were associated with markedly reduced risks (approximately 38-84%) of DR onset and progression, as well as with more favorable retinal structural parameters (250). Collectively, these data support a consistent inverse association between PA and DR, while underscoring the need for mechanistically anchored human studies.

## Circulating exerkines in DR: strengths and gaps in human evidence

**Figure 5:**
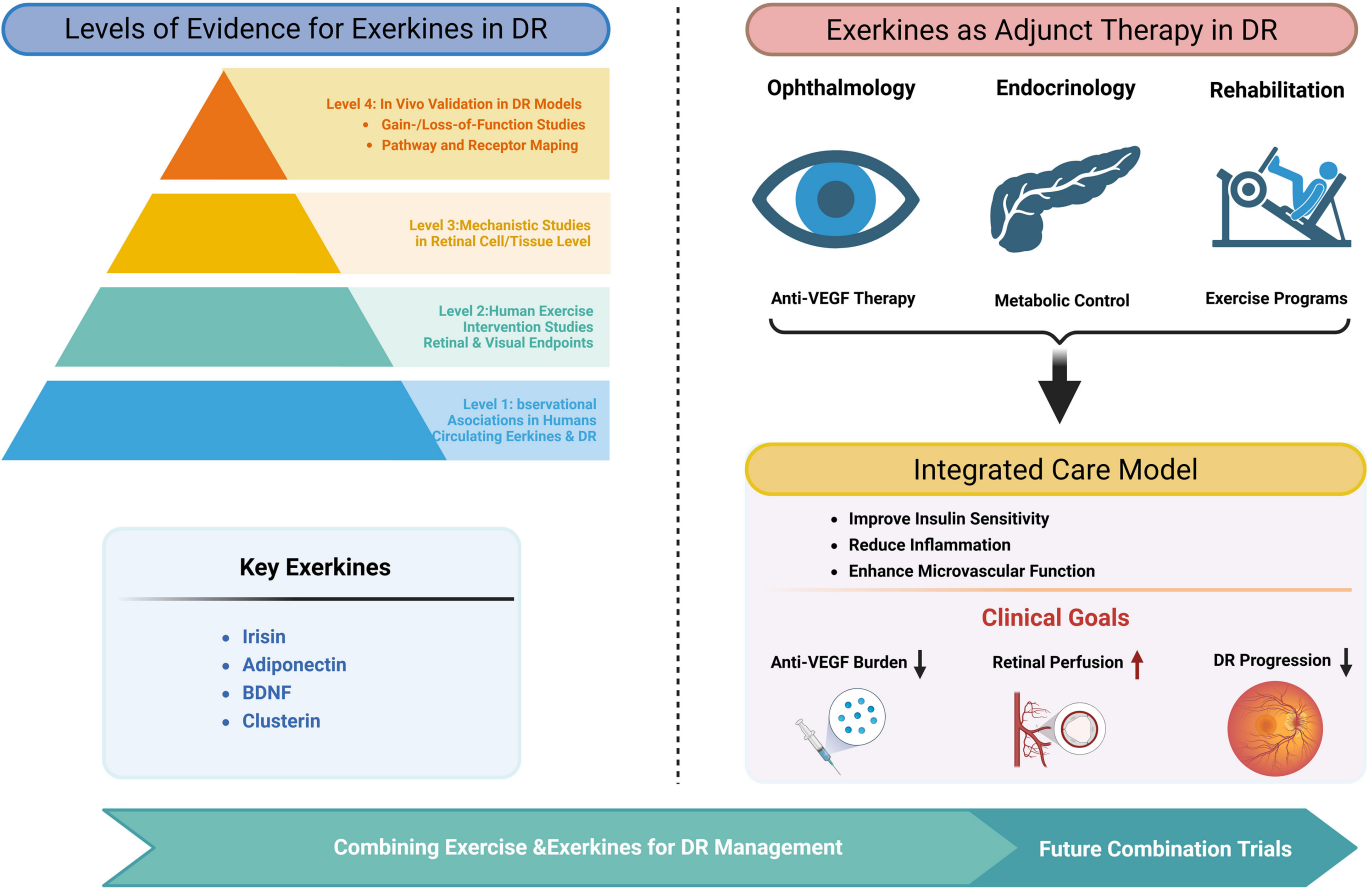
Conceptual framework for the evidence hierarchy and clinical integration of exerkines in DR. The left panel illustrates a pyramidal hierarchy of evidence supporting the role of exerkines in DR, ranging from Level 1 observational associations between circulating exerkines and DR in humans, to Level 2 human exercise intervention studies assessing retinal and visual outcomes, Level 3 mechanistic studies at the retinal cell and tissue level, and Level 4 *in vivo* validation in DR models including gain- and loss-of-function approaches and pathway/receptor mapping. Key candidate exerkines highlighted include irisin, adiponectin, BDNF, and clusterin. The right panel depicts exerkines as an adjunct therapeutic strategy in DR, integrating anti-VEGF therapy, metabolic control, and structured exercise programs into an integrated care model. This multidisciplinary approach is proposed to improve insulin sensitivity, reduce inflammation, and enhance retinal microvascular function, ultimately lowering anti-VEGF treatment burden, improving retinal perfusion, and slowing DR progression.

To provide a transparent appraisal of the current state of the exerkine-DR field, we propose a structured “levels of evidence” framework encompassing four tiers: (i) observational associations between circulating exerkines and DR in humans; (ii) human exercise intervention studies incorporating retinal or visual endpoints; (iii) mechanistic investigations in retinal cells or tissues; and (iv) causal *in vivo* validation in diabetic models, including gain- and loss-of-function approaches and receptor or pathway mapping. Application of this framework to leading candidate exerkines reveals a highly uneven evidentiary landscape, with robust support at select levels but substantial gaps in causal and mechanistic validation (**Figure 5**).

### Irisin

Irisin shows the most consistent inverse relationship with DR. Early studies demonstrated differences in serum and vitreous irisin levels between diabetic patients with and without microvascular complications, including DR (251). Subsequent work confirmed that circulating irisin is significantly lower in patients with type 2 diabetes and chronic complications such as nephropathy and/or retinopathy, with levels declining in parallel with complication burden (220, 241). Importantly, a recent DR-focused clinical study reported higher serum irisin levels in patients without retinopathy compared with those with DR, even after adjustment for HbA1c and diabetes duration, supporting a potential protective or compensatory role (252). Key limitations include assay variability and uncertainty regarding biologically active irisin species.

### Adiponectin

Adiponectin exhibits context-dependent associations with DR. Several cohorts report positive correlations between total or high-molecular-weight adiponectin levels and DR severity in type 2 diabetes (246). In contrast, mechanistic and translational studies emphasize adiponectin’s protective actions on insulin resistance, oxidative stress, inflammation, and modulation of the AGE–RAGE axis in the diabetic retina (216, 253). Renal dysfunction, obesity, and advanced disease stage likely confound circulating adiponectin levels, complicating its interpretation as a linear biomarker (216, 246, 253).

### BDNF

BDNF demonstrates relatively consistent inverse associations with DR. Clinical studies and meta-analyses report significantly reduced circulating BDNF levels in patients with DR, particularly vision-threatening forms, compared with diabetic patients without retinopathy (238, 254). A recent clinical study proposed BDNF as an emerging biomarker for DR and suggested that enhancing BDNF signaling could represent a therapeutic strategy aligned with exercise-induced neuroprotection (162, 245).

### Clusterin

Clusterin is supported primarily by experimental and histologic evidence. Increased clusterin expression has been observed in diabetic retinas, particularly around vascular lesions, and functional studies demonstrate that clusterin can attenuate BRB disruption and neural retinal injury in early experimental diabetes (238, 239, 245). Although large-scale human circulating data are limited, clusterin is known to be modulated by metabolic status and physical training in other systems, nominating it as a plausible exerkine candidate in DR that warrants targeted biomarker studies (239). Overall, human data indicate that exercise-responsive circulating factors track with DR presence and severity, but are limited by cross-sectional design, modest sample sizes, assay heterogeneity, and incomplete control of confounding variables.

## Exerkines as adjunct therapy in DR: clinical integration

Anti-VEGF therapy remains central to DR management but does not directly address systemic metabolic and inflammatory drivers of disease and is associated with a substantial real-world treatment burden and adherence challenges (255, 256). Exerkines may complement current therapies by improving insulin sensitivity, reducing chronic inflammation, and enhancing endothelial and microvascular function (23, 257). A practical translational approach involves coordinated ophthalmology-endocrinology-rehabilitation care pathways, in which structured exercise programs are integrated with routine ocular and metabolic management. In this model, ophthalmologists guide anti-VEGF therapy, endocrinologists optimize metabolic control, and rehabilitation specialists tailor exercise prescriptions consistent with diabetes exercise guidance (including precautions in advanced/unstable retinopathy) (119). Exerkine-mediated systemic benefits may synergize with local anti-angiogenic effects, potentially improving retinal perfusion and stabilizing the BRB; population data also link higher physical activity to lower DR progression risk (258). These effects raise the possibility that regular exercise could reduce anti-VEGF injection frequency or prolong treatment intervals, a hypothesis that is now testable in pragmatic combination trials (255, 256). Future trials should assess anti-VEGF injection burden, OCT and OCTA-based structural/perfusion outcomes, and circulating metabolic and exerkine biomarkers to define the clinical value of this integrative strategy (255, 259).

## Challenges, knowledge gaps, and future directions

Despite rapid progress in exerkine biology, direct causal links between individual exercise-induced factors and DR remain largely speculative, particularly in humans. Much of the current mechanistic understanding of exerkines such as irisin, IL-6, myonectin, GDF15, and clusterin derives from cardiometabolic, neurological, or renal contexts rather than retinal systems (132, 260, 261). Although epidemiological and interventional studies consistently report that physical activity is associated with reduced DR incidence or slower disease progression (21, 258, 262), very few human studies have directly interrogated whether specific exercise-induced exerkines mediate these protective effects, representing a critical translational gap. As a result, it remains unclear whether candidate exerkines act directly on retinal neurons, glia, or vascular cells, indirectly through systemic metabolic and inflammatory improvements, or merely serve as biomarkers of broader physiological adaptation to exercise.

Preclinical studies using STZ-induced diabetes, db/db mice, or high-fat diet–STZ combination models provide important proof-of-concept that exercise can attenuate retinal inflammation, oxidative stress, vascular leakage, and neurodegeneration (21, 263, 264). However, these models have inherent limitations for translational inference that are insufficiently appreciated. Rodent retinas differ from human retinas in vascular architecture, photoreceptor distribution, metabolic demand, and susceptibility to ischemia, and disease progression in rodents is compressed relative to the decades-long course of human DR. Moreover, systemic metabolic responses to exercise, including muscle mass, fiber composition, and exerkine secretion dynamics, differ substantially between rodents and humans, raising uncertainty about dose-response relationships and temporal signaling. Importantly, most animal studies manipulate exercise behavior rather than individual exerkines, making it difficult to distinguish whether retinal benefits arise from specific circulating mediators, upstream regulators such as AMPK or PGC-1α, or secondary metabolic improvements. These species- and model-specific constraints underscore the need for careful validation of exerkine-retina mechanisms in human-relevant systems.

Methodological heterogeneity further complicates interpretation across studies. Current investigations employ diverse analytical platforms, including ELISA, multiplex immunoassays, mass spectrometry-based proteomics, and targeted metabolomics, each with distinct sensitivity, specificity, and dynamic range (132, 260, 261, 265). Variability in pre-analytical factors (fasting status, timing relative to exercise, anticoagulant choice, and storage conditions) and inconsistent unit reporting introduce additional noise. Exercise interventions in DR populations vary widely in modality, intensity, duration, adherence, and co-interventions (21, 258, 262), while retinal endpoints range from fundus-based grading to OCT-derived central macular thickness (CMT) and functional measures. Notably, only a small number of randomized trials have incorporated standardized exercise regimens with OCT-based endpoints, and these studies generally lack parallel profiling of circulating exerkines (266), limiting mechanistic interpretation.

Addressing these challenges will require well-powered, rigorously designed randomized controlled trials in diabetic patients that integrate standardized exercise prescriptions with longitudinal exerkine profiling and validated retinal outcomes, including ETDRS grading, CMT, OCT angiography, microperimetry, and visual performance. Harmonized biospecimen protocols and predefined exercise dosing will be essential to capture exerkine kinetics and reduce technical noise. Such designs are critical to determine whether exerkines function as causal mediators, permissive modifiers, or epiphenomena of exercise-induced metabolic health.

Emerging technologies offer promising avenues to bridge preclinical and clinical research. Single-cell and single-nucleus RNA sequencing has begun to define cell type-specific transcriptional states across DR stages in retinal endothelial cells, pericytes, Müller glia, microglia, and neurons, as well as circulating immune populations linked to retinal pathology (267–270). Integrating these datasets with plasma proteomics and metabolomics from exercise-intervened diabetic cohorts could reveal receptor-ligand interactions connecting exerkines to retinal signaling pathways and identify convergent “exerkine nodes” within inflammatory, fibrotic, or angiogenic networks (271). Advanced human-relevant platforms, including vascularized retinal organoids, retina-on-chip, and blood-retina barrier-on-chip systems, enable controlled testing of exerkine cocktails, mimetics, or gene therapies under physiologically relevant flow, glucose, and oxygen gradients (263, 264, 272, 273).

To move beyond association, future studies should prioritize exercise-intervention trials incorporating retinal endpoints alongside serial exerkine measurements. In parallel, pharmacologic “exercise mimetics” offer translational momentum. Long-acting FGF21 analogs now in clinical development for metabolic liver disease (MASH/NASH) and related metabolic indications, have documented anti-inflammatory and vascular/endothelial-protective actions that are plausibly relevant to retinal microvasculature (274, 275). Consistent with this rationale, long-acting FGF21 has been shown to inhibit retinal vascular leakage in experimental settings (276), and FGF21 signaling can suppress pathological retinal/choroidal neovascularization in preclinical models (218). In addition, stabilized/long-acting irisin formats are under active preclinical development to address the short half-life and instability of native irisin, and pharmacologic targeting of the FNDC5/irisin axis is facilitated by identification of αV-class integrins (including αV/β5) as irisin receptors (277, 278). Although ocular-specific studies remain limited, these approaches highlight the feasibility of therapeutically targeting exerkine pathways to recapitulate exercise-induced retinal protection.

To most effectively advance the field, future research must move beyond association to establish whether specific exerkines causally mediate retinal protection in humans with diabetes or instead function as surrogate markers of systemic metabolic improvement. This effort will require defining the exercise dose, modality, and temporal pattern that most effectively modulate exerkine profiles relevant to retinal health, as well as identifying the retinal cell types, endothelial cells, pericytes, Müller glia, or neurons, that directly respond to candidate exerkines in human-relevant systems. In parallel, translational studies must determine whether exerkine-targeted therapies or exercise mimetics can safely and reproducibly recapitulate the retinal benefits of physical activity, while accounting for inter-individual variability driven by age, disease stage, and metabolic status that may influence exerkine responsiveness and therapeutic feasibility. Ultimately, a coordinated translational roadmap integrating longitudinal human cohorts, mechanistic organoid and organ-on-chip models, systems biology pipelines, and staged clinical trials will be required to move the field beyond correlation, clarify whether exerkines represent viable therapeutic targets or mechanistic biomarkers, and ensure that emerging pharmacological strategies complement, rather than replace, exercise-based interventions that remain foundational in the prevention and management of diabetic retinopathy (21, 279) (**Table 3**).

**Table 3:** Key sources of methodological heterogeneity in exerkine research and their impact on retinal outcomes.

Domain	Major sources of variability	Implications
Study design	Cross-sectional vs longitudinal; observational vs interventional	Limits causal inference
Species/models	Rodents vs humans; STZ vs db/db vs HFD–STZ	Restricted translational relevance
Exercise protocols	Modality, intensity, duration, adherence	Poor comparability across trials
Exerkine assays	ELISA vs multiplex vs proteomics; timing of sampling	Inconsistent quantification
Pre-analytical factors	Fasting status, anticoagulant, storage	Measurement noise
Retinal outcomes	Fundus grading, OCT, OCTA, functional tests.	Variable sensitivity to change.

## Conclusion

Accumulating evidence suggests that exercise-induced exerkines contribute to retinal protection in diabetes by modulating inflammation, oxidative stress, neurovascular integrity, metabolic homeostasis, and neuronal survival within the retinal neurovascular unit. These effects are not mediated by a single pathway but arise from coordinated, cell type- and context-dependent actions that vary with exercise modality and disease stage. This review presents a retina-centered, cell-resolved framework integrating exerkine biology with core mechanisms of diabetic retinopathy, while explicitly distinguishing retinal-specific evidence from hypotheses derived from non-ocular systems. By linking exercise-dependent exerkine signatures to distinct retinal processes, we identify potential therapeutic implications as well as important biological and safety limitations. At present, exerkines should be viewed as candidate mediators, modifiers, or biomarkers of exercise-induced retinal benefit rather than established therapeutic agents. Translation to clinical practice will require well-designed human studies combining standardized exercise interventions, longitudinal exerkine profiling, and validated retinal endpoints, together with advances in human-relevant experimental models and pharmacological exercise mimetics. Integrating lifestyle-based interventions with mechanistically informed molecular research may ultimately provide a rational strategy to address the multifactorial pathogenesis of diabetic retinopathy.
